# Nicotine dependence and self-esteem among respiratory therapy students in Saudi Arabia: A cross-sectional study

**DOI:** 10.18332/tid/221536

**Published:** 2026-06-17

**Authors:** Abdullah S. Alsulayyim, Rami A. Alyami, Abdulaziz S. Al Sharifi, Ali Hakamy, Ali M. Alasmari, Ziyad Alshehri, Fahad H. Alahmadi, Saeed M. Alghamdi, Mohammed A. Almeshari, Abdulrhman S. Alghamdi, Mohammed M. Alyami, Jaber S. Alqahtani, Nowaf Y. Alobaidi, Bedor S. Alkhathlan, Khaled A. Alqahtani, Hooriah H. Alowiwi, Fahad H. Algharbi, Abdullah A. Alqarni, Abdulelah M. Aldhahir

**Affiliations:** 1 Respiratory Therapy Program, Department of Nursing, College of Nursing and Health Sciences, Jazan University, Jizan, Saudi Arabia; 2 Respiratory Therapy Department, College of Medical Rehabilitation Sciences, Taibah University, Madinah, Saudi Arabia; 3 Clinical Technology Department, Respiratory Care Program, Faculty of Applied Medical Sciences, Umm Al-Qura University, Makkah, Saudi Arabia; 4 Rehabilitation Health Sciences Department, College of Applied Medical Sciences, King Saud University, Riyadh, Saudi Arabia; 5 Respiratory Therapy Department, Batterjee Medical College, Khamis Mushait, Saudi Arabia; 6 Department of Respiratory Care, Prince Sultan Military College of Health Sciences, Dammam, Saudi Arabia; 7 Department of Respiratory Therapy, College of Applied Medical Sciences, King Saud Bin Abdulaziz University for Health Sciences, Alahsa, Saudi Arabia; 8 King Abdullah International Medical Research Center, Alahsa, Saudi Arabia; 9 Department of Respiratory Care Services, King Abdulaziz Hospital, Ministry of National Guard-Health Affairs, Al Ahsa, Saudi Arabia; 10 Medical Support Services, Royal Commission Health Services Program, Royal Commission for Jubail and Yanbu, Jubail, Saudi Arabia; 11 Department of Physiotherapy, College of Imam Abdulrahman bin Faisal University, Dammam, Saudi Arabia; 12 Department of Respiratory Therapy, Faculty of Medical Rehabilitation Sciences, King Abdulaziz University, Jeddah, Saudi Arabia; 13 Respiratory Therapy Unit, King Abdulaziz University Hospital, Jeddah, Saudi Arabia

**Keywords:** nicotine dependence, selfesteem, respiratory therapy students, Saudi Arabia, smoking

## Abstract

**INTRODUCTION:**

Nicotine dependence among university students remains a public health concern, and psychological factors such as self-esteem may influence dependence severity. Evidence among respiratory therapy students in Saudi Arabia is limited. Hence, this study aimed to assess the relationship between nicotine dependence and self-esteem among respiratory therapy students in Saudi Arabia.

**METHODS:**

This cross-sectional study was conducted between 13 March and 16 September 2025, and included 1041 respiratory therapy students. Inclusion criteria were enrollment from the second academic year through the internship year; first-year students were excluded. Data were collected via an anonymous online survey assessing smoking history, nicotine dependence using the Fagerström test for nicotine dependence (FTND), and self-esteem using the Rosenberg Self-Esteem Scale (RSES). Negative binomial regression was used to identify factors associated with nicotine dependence.

**RESULTS:**

The median FTND score was 5 (IQR: 3–6). Smoking duration was significantly associated with higher nicotine dependence in a dose–response pattern. Compared with students who smoked for less than one year, those with 1–5 years (adjusted incidence rate ratio, IRR=1.10; 95% CI: 1.03–1.19; p=0.008), 6–10 years (IRR=1.15; 95% CI: 1.04–1.27; p=0.006), and >10 years of smoking (IRR = 1.31; 95% CI: 1.07–1.60; p=0.009) had higher dependence scores. Higher self-esteem was independently associated with lower nicotine dependence, with each one-point increase in RSES score linked to a 3% reduction in FTND score (IRR=0.97; 95% CI: 0.95–0.99; p=0.011). No demographic or academic variables were significantly associated with nicotine dependence.

**CONCLUSIONS:**

Nicotine dependence among respiratory therapy students is strongly related to smoking duration and self-esteem. Further longitudinal and interventional studies are needed to clarify the temporal relationship between selfe-steem and nicotine dependence and to determine whether psychosocial support could complement tobacco control efforts in university settings.

## INTRODUCTION

Due to growing social, psychological, and cultural norms that could encourage tobacco use in early adulthood, nicotine dependence is a serious health concern among university students worldwide^1,2^, including those in Saudi Arabia^3^. Notably, a complex interplay of factors, including peer pressure, stress, accessibility, and underlying mental health conditions, could drive the prevalence of tobacco product use among Saudi university students^3^. While overall smoking rates may vary, recent studies have shown that the use of electronic cigarettes is becoming more widespread and indicates a changing trend in this population’s patterns of nicotine consumption^4,5^. Another trend that is currently being seen and studied is the use of nicotine pouches as a substitute or in addition to traditional cigarette smoking^6^.

Nicotine addiction is acknowledged as a significant contributor to the worldwide burden of disease, causing millions of deaths and substantial morbidity annually^2,7^. In 2021, smoking-related disability-adjusted life years (DALYs) totaled over 160 million, or approximately 6% of the global DALY burden, while smoking-attributable deaths reached over 6 million worldwide, accounting for more than 9% of all deaths^8^. Beyond cancer and respiratory conditions, nicotine addiction has a wide range of cardiovascular, neurological, and metabolic problems that worsen the risk of early death and shorter life expectancy^8^.

Nicotine dependence has a significant societal impact, as evidenced by the fact that it is associated with annual global costs exceeding one trillion US dollars due to healthcare expenses and lost productivity^9^. Even though traditional smoking rates have decreased in some areas, the overall number of nicotine users is still high, partly due to new nicotine delivery methods, including e-cigarettes^10^. Given the ongoing and growing toll that tobacco and nicotine use have on health systems and societies around the world, the body of evidence highlights the urgent need for increased public health initiatives to reduce tobacco and nicotine use^8^.

Understanding the psychological association of nicotine dependence, particularly self-esteem, is critical, as both constructs are closely linked to students’ emotional and social well-being^11^. Research indicated that psychological discomfort, including symptoms such as low self-esteem, is linked to a higher susceptibility to nicotine dependence, and people often use nicotine as a coping mechanism to deal with emotional distress or problems with their self-concept^12,13^. Mental health issues and familial or social factors can exacerbate this relationship by raising the risk of substance abuse^12^.

The Fagerström test for nicotine dependence (FTND) ^14^ is a widely used instrument for assessing nicotine dependence, while the Rosenberg Self-Esteem Scale (RSES)^15^ is commonly used to evaluate global self-esteem. Both the FTND and RSES have been widely validated across diverse populations. The FTND demonstrates reliable performance in different cultural set-

tings^16,17^, while the RSES shows strong cross-cultural validity and measurement invariance^18^. Given these considerations, the current study aims to assess the relationship between nicotine dependence and self-esteem among respiratory therapy students in Saudi Arabia.

## METHODS

### Study design and setting

This cross-sectional study was conducted between 13 March and 16 September 2025, to assess the association between nicotine dependence and self-esteem among respiratory therapy students in Saudi Arabia.

### Participants and sampling

The study included 1041 respiratory therapy students, recruited through convenience sampling. Electronic informed consent was obtained from all participants prior to participation. The inclusion criteria were current smoker students from the second academic year through the internship year. First-year (pre-medical) students were excluded because they had not yet begun the core respiratory therapy curriculum. Ethical approval for this study was obtained from the Standing Committee for Scientific Research at Jazan University (Reference No. REC-45/02/740).

### Data collection

Data were collected using the SurveyMonkey platform between 13 March and 16 September 2025, through an anonymous online survey distributed via platforms such as WhatsApp, X, etc. The survey included three sections: 1) demographic and academic information, including age, gender, smoking history, monthly income, university sector, cumulative grade point average (GPA), academic level, absenteeism, and academic warnings; 2) nicotine dependence assessment using the FTND; and 3) self-esteem assessment using the RSES. Nicotine dependence was the primary outcome, measured as a continuous FTND score (0–10), and self-esteem was the primary exposure, measured as a continuous RSES score (0–30). The FTND comprises six items assessing key smoking behaviors, including time to first cigarette, smoking frequency, and dependence-related habits, using predefined categorical responses. The RSES includes 10 items measuring global self-esteem, with responses on a four-point Likert scale (strongly disagree to strongly agree), incorporating both positively and negatively worded items, the latter of which are reverse-coded. Age was treated as a continuous variable, while gender (male, female), smoking history (<1, 1–5, 6–10, >10 years), monthly income (<1000, 1000–3000, >3000 Saudi Riyals [SAR]), university sector (governmental, private), cumulative Grade Point Average (2.00–2.99, 3.00–3.99, 4.00–5.00), academic level (first year, second year, third year, fourth year, bridging, intern), absenteeism (none, 1–2, 3–4, 5–6, >6 days), and academic warnings (none, 1, 2, 3, >3 warnings) were categorized as collected.

### Sample size

Drawing on a previously published study of respiratory therapy (RT) students in Saudi Arabia (n=1297)^19^, and considering the expected rise to nearly 3000 students following the introduction of new programs, the estimated sample size ranged from 341 (with a 5% margin of error) to 1017 (with a 2.5% margin of error). In 2025, we recruited 1041 RT students, surpassing the upper estimate and thereby ensuring sufficient statistical power and precision for the analysis.

### Measures

Nicotine dependence was assessed using the FTND^14^. The FTND comprises six items evaluating key aspects of smoking behavior, including daily cigarette consumption, nicotine dependence, and difficulty refraining from smoking in specific situations. Item responses were scored according to the FTND scoring system and summed to generate a total FTND score for each participant. FTND scores were also categorized into four dependence levels based on total score: low dependence (1–2), low-to-moderate dependence (3–4), moderate dependence (5–7), and high dependence (≥8). The FTND is widely regarded as reliable in both clinical and research settings, with established associations with withdrawal symptoms, craving intensity, and other markers of addiction^20^.

Self-esteem was assessed using the RSES, a widely used and validated instrument for measuring global self-esteem across diverse populations. Although originally conceptualized as unidimensional, factor analytic studies have identified both positive and negative item dimensions. The scale demonstrates strong psychometric properties, including high internal consistency and cross-cultural validity, and has been widely applied in student populations^15,18^. For scoring, positively worded items were assigned as: strongly agree=3, agree=2, disagree=1, and strongly disagree=0. Negatively worded items (At times I think I am no good at all; I feel I do not have much to be proud of; I wish I could have more respect for myself; I certainly feel useless at times; All in all, I am inclined to think that I am a failure) were reverse-scored (strongly agree=0; agree=1; disagree=2; strongly disagree=3). Item scores were summed to obtain a total RSES score (0–30), with higher scores indicating higher self-esteem. No cutoff was applied; self-esteem was treated as a continuous variable in inferential analyses.

### Statistical analysis

Data were analyzed using Stata/SE 18. Demographic characteristics were summarized as frequencies and percentages for categorical variables and means with standard deviations (SD) and median and IQR for continuous variables. To identify factors associated with nicotine dependence, multivariable negative binomial regression was performed with FTND total score as the dependent variable. Results are reported as adjusted incidence rate ratios (IRRs) with 95% confidence intervals (CIs). The RSES score was included in the model as a continuous independent variable. Categorical predictors (gender, smoking history, monthly income, university sector, cumulative GPA, academic level, absenteeism category, and academic warnings) were included as factor variables, with reference categories selected based on subgroup size or conceptual relevance. These variables were treated as potential confounders in the multivariable regression analysis. Statistical significance was set at p<0.05. Missing data were handled using complete-case analysis. As the study used convenience sampling, no sampling weights were applied; therefore, the results should be interpreted with consideration of the non-probability sampling design.

## RESULTS

### Participant characteristics

Demographic and academic characteristics of the participants are summarized in Table 1. A total of 1041 participants were included in the analysis. Most participants were male (635; 61.0%). The mean age was 21.35 ± 1.60 years. Most participants reported a smoking history of 1–5 years (467; 44.9%) or <1 year (401; 38.5%), 154 (14.8%) reported 6–10 years, and 19 (1.8%) reported >10 years. Monthly income (SAR) was most commonly <1000 (455; 43.7%), followed by 1000–3000 (386; 37.1%) and >3000 (200; 19.2%). Most participants were enrolled in governmental universities (796; 76.5%) compared with private universities (245; 23.5%) (Table 1).

**Table 1:** Characteristics of respiratory therapy students in Saudi Arabia, a cross-sectional study conducted March – September 2025 (N=1041)

*Characteristics*	*n (%)*
**Gender**	
Female	406 (39.0)
Male	635 (61.0)
**Age** (years), mean ± SD	21 ± 1.60
**Smoking history** (years)	
<1	401 (38.5)
1–5	467 (44.9)
6–10	154 (14.8)
>10	19 (1.8)
**Monthly income** (SAR)	
<1000	455 (43.7)
1000–3000	386 (37.1)
>3000	200 (19.2)
**University sector**	
Governmental	796 (76.5)
Private	245 (23.5)
**Cumulative grade point average** (GPA)	
2.00–2.99	40 (3.8)
3.00–3.99	442 (42.5)
4.00–5.00	559 (53.7)
**Academic level**	
First year	26 (2.5)
Second year	322 (30.9)
Third year	365 (35.1)
Fourth year	248 (23.8)
Bridging	5 (0.5)
Intern	75 (7.2)
**Days absent from classes during last semester** (days)	
0	321 (30.8)
1–2	300 (28.8)
3–4	353 (33.9)
5–6	58 (5.6)
>6	9 (0.9)
**Academic warnings received**	
0	400 (38.4)
1	342 (32.9)
2	257 (24.7)
3	38 (3.7)
>3	4 (0.4)

SAR: 1000 Saudi Riyals about US$270.

Regarding academic performance, 559 (53.7%) had a GPA of 4.00–5.00, 442 (42.5%) had 3.00–3.99, and 40 (3.8%) had 2.00–2.99. The most common academic levels were third year (365; 35.1%) and second year (322; 30.9%), followed by fourth year (248; 23.8%), interns (75; 7.2%), first year (26; 2.5%), and bridging (5; 0.5%). Regarding attendance, 321 (30.8%) reported no absences in the last semester, while 353 (33.9%) reported 3–4 days absent. For academic warnings, 400 (38.4%) reported no warnings, and 342 (32.9%) reported one warning (Table 1).

### Nicotine dependence

Responses to individual items of the FTND are summarized in Table 2. Overall, the median FTND score (IQR) was 5 (3–6), with scores ranging from 1 to 10. Half of participants reported smoking their first cigarette 31–60 minutes after waking (50.0%), while 26.9% reported smoking within 5–30 minutes, and 23.2% within 1 minute. Nearly half of the participants reported difficulty refraining from smoking in places where smoking is forbidden (46.4%). When asked which cigarette they would hate most to give up, 44.0% selected the first cigarette in the morning, whereas 56.0% selected any other cigarette.

**Table 2:** Nicotine dependence item responses of respiratory therapy students in Saudi Arabia, a crosssectional study conducted March – September 2025 (N=1041)

*Questions*	*n (%)*
**How soon after waking do you smoke your 1st cigarette?** (minutes)	
1	241 (23.2)
31–60	520 (50.0)
5–30	280 (26.9)
**Do you find it difficult to refrain from smoking in places where it is forbidden?**	
No	558 (53.6)
Yes	483 (46.4)
**Which cigarette would you hate to give up?**	
Any other	583 (56.0)
The first in the morning	458 (44.0)
**How many cigarettes do you smoke per day?**	
≤10	461 (44.3)
11–20	360 (34.6)
21–30	182 (17.5)
≥31	38 (3.7)
**Do you smoke more frequently in the morning?**	
No	525 (50.4)
Yes	516 (49.6)
**Do you smoke even if you are sick in bed most of the day?**	
No	561 (53.9)
Yes	480 (46.1)

Regarding daily consumption, 461 (44.3%) smoked 10 cigarettes or fewer per day, 360 (34.6%) smoked 11–20, 182 (17.5%) smoked 21–30, and 38 (3.7%) smoked ≥31. About half reported smoking more frequently in the morning (516; 49.6%), and 480 (46.1%) reported smoking even when sick in bed most of the day (Table 2).

### Self-esteem

Responses to the RSES items are presented in Table 3. Overall, the median RSES score was 15 (IQR: 14–15) with scores ranging 9–23. For the positively worded statement: ‘On the whole, I am satisfied with myself’, most participants selected disagree (543; 52.2%) or strongly disagree (249; 23.9%), while 227 (21.8%) agreed and 22 (2.1%) strongly agreed. A similar pattern was observed for other positively worded items, including: ‘I feel that I have a number of good qualities’ (disagree: 500, 48.0%; strongly disagree: 349, 33.5%) and ‘I take a positive attitude toward myself’ (disagree: 511, 49.1%; strongly disagree: 327, 31.4%) (Table 3).

**Table 3:** Rosenberg Self-Esteem Scale item responses of respiratory therapy students in Saudi Arabia, a crosssectional study conducted March – September 2025 (N=1041)

*RSES item*	*Strongly disagree* *n (%)*	*Disagree* *n (%)*	*Agree* *n (%)*	*Strongly agree* *n (%)*
On the whole, I am satisfied with myself	249 (23.9)	543 (52.2)	227 (21.8)	22 (2.1)
At times I think I am no good at all	128 (12.3)	55 (5.3)	367 (35.3)	491 (47.2)
I feel that I have a number of good qualities	349 (33.5)	500 (48.0)	131 (12.6)	61 (5.9)
I am able to do things as well as most other people	351 (33.7)	482 (46.3)	160 (15.4)	48 (4.6)
I feel I do not have much to be proud of	116 (11.1)	99 (9.5)	403 (38.7)	423 (40.6)
I certainly feel useless at times.	149 (14.3)	83 (8.0)	355 (34.1)	454 (43.6)
I feel that I’m a person of worth, at least on an equal plane with others	360 (34.6)	425 (40.8)	159 (15.3)	97 (9.3)
I wish I could have more respect for myself	126 (12.1)	73 (7.0)	341 (32.8)	501 (48.1)
All in all, I tend to feel that I am a failure	140 (13.4)	65 (6.2)	363 (34.9)	473 (45.4)
I take a positive attitude toward myself	327 (31.4)	511 (49.1)	139 (13.4)	64 (6.1)

In contrast, endorsement of negatively worded items was common. For ‘At times I think I am no good at all’, 491 (47.2%) strongly agreed, and 367 (35.3%) agreed. Likewise, for: ‘All in all, I tend to feel that I am a failure’, 473 (45.4%) strongly agreed, and 363 (34.9%) agreed. High agreement was also observed for ‘I wish I could have more respect for myself’, where 501 (48.1%) strongly agreed, and 341 (32.8%) agreed. Comparable response patterns were seen for other negative items, such as ‘I certainly feel useless at times’ (strongly agree: 454, 43.6%; agree: 355, 34.1%) and ‘I feel I do not have much to be proud of’ (strongly agree: 423, 40.6%; agree: 403, 38.7%) (Table 3).

### Self-esteem and other factors associated with nicotine dependence (multivariable analysis)

A negative binomial regression model was fitted to examine factors associated with nicotine dependence as measured by the Fagerström score (Table 4). After adjustment for covariates, smoking history was significantly associated with nicotine dependence. Compared with participants who reported smoking for <1 year, those who smoked for 1–5 years (IRR=1.103; 95% CI: 1.026–1.186; p=0.008), 6–10 years (IRR=1.148; 95% CI: 1.041–1.267; p=0.006), and >10 years (IRR=1.305; 95% CI 1.068–1.596; p=0.009) had significantly higher expected nicotine dependence scores, demonstrating a dose–response pattern.

**Table 4:** Multivariable negative binomial regression predicting nicotine dependence, measured by the continuous Fagerström test for nicotine dependence score among respiratory therapy students in Saudi Arabia, a cross-sectional study conducted March – September 2025 (N=1041)

*Variables*	*Adjusted IRR*	*95% CI*	*p*
**Gender** (ref. female)			
Male	1.018	0.959–1.081	0.557
**Smoking history** (ref. <1 year)			
1–5	1.103	1.026–1.186	0.008
6–10	1.148	1.041–1.267	0.006
>10	1.305	1.068–1.596	0.009
**Monthly income** (ref. <1000 SAR)			
1000–3000	1.014	0.943–1.091	0.701
>3000	0.961	0.878–1.052	0.389
**University sector** (ref. governmental)			
Private	1.022	0.955–1.095	0.525
**Cumulative GPA** (ref. 2.00–2.99)			
3.00–3.99	0.882	0.765–1.017	0.084
4.00–5.00	0.882	0.765–1.018	0.085
**Academic level** (ref. first year)			
Second year	1.117	0.927–1.347	0.245
Third year	1.115	0.924–1.344	0.256
Fourth year	0.946	0.781–1.147	0.573
Bridging	1.071	0.661–1.735	0.780
Intern	1.026	0.829–1.270	0.814
**Absent days** (ref. none)			
1–2	0.957	0.859–1.067	0.430
3–4	0.912	0.811–1.026	0.126
5–6	0.974	0.830–1.142	0.742
>6	0.798	0.563–1.129	0.202
**Academic warnings** (ref. none)			
1	1.028	0.929–1.136	0.598
2	1.059	0.950–1.180	0.300
3	1.148	0.969–1.360	0.111
>3	1.226	0.787–1.909	0.368
**Self-esteem** (RSES total score)	0.972	0.952–0.993	0.011

IRR: incidence rate ratio. RSES: Rosenberg Self-Esteem Scale. SAR: 1000 Saudi Riyals about US$270.

Self-esteem (Rosenberg Self-Esteem Scale total score) was also statistically significantly associated with nicotine dependence. Each one-point increase in the RSES score was associated with a 3% lower expected nicotine dependence score (IRR=0.972; 95% CI 0.952–0.993; p=0.011). No other covariates (gender, monthly income, university sector, cumulative GPA, academic level, absenteeism, or academic warnings) were statistically significant in the adjusted model (all p>0.05) (Table 4).

## DISCUSSION

In this study, nicotine dependence was characterized by patterns consistent with early-stage smoking behavior, while self-esteem levels were generally low. A clear dose-response relationship was observed between smoking duration and nicotine dependence, and higher self-esteem was independently associated with lower dependence. In contrast, demographic and academic variables were not significant predictors after adjustment.

Exposure to smoking during early to moderate durations represents a critical period for the development of tobacco-related harm and highlights opportunities for prevention. Even relatively low-intensity smoking has been associated with early physiological changes, including inflammatory and oxidative processes affecting lung function^21,22^. Social and environmental influences, particularly peer dynamics and cultural context, are also important determinants of smoking initiation and continuation in university populations^23,24^. These findings support a biopsychosocial framework in understanding nicotine dependence and its progression^25,26^.

Dependence-related behaviors observed in this study, such as difficulty abstaining in restricted settings and increased smoking during specific periods, are consistent with established features of nicotine addiction. These behaviors reflect impaired control over tobacco use and prioritization of craving relief over social or environmental constraints^27^. Established markers of dependence, including early smoking after waking and increased consumption patterns, are closely linked to nicotine dependence severity^28^. The interplay of biological, psychological, and social influences further underscores the complexity of nicotine dependence in this population^27,28^.

Self-esteem patterns in this study suggest a tendency toward negative self-evaluation. This differs from findings in large cross-national studies, where overall self-esteem tends to be more positive across populations^18^. However, this pattern may partly reflect methodological effects related to item wording, as differences between positively and negatively phrased items can introduce systematic bias^29^. Cultural response styles may also influence how individuals interpret and respond to self-report scales, complicating direct comparisons across populations^21^. The observed inverse association between self-esteem and nicotine dependence is consistent with prior research showing that lower self-esteem is linked to higher dependence and increased likelihood of smoking initiation and persistence^13,30^. The relationship between nicotine dependence and self-esteem may be bidirectional; however, the cross-sectional design of this study precludes determination of causality or directionality.

The lack of independent associations between nicotine dependence and demographic or academic variables suggests that these factors may be more relevant to smoking initiation than to dependence severity once smoking is established. This aligns with previous studies indicating that gender differences in smoking behavior do not necessarily translate into differences in dependence levels^31^. Similarly, socioeconomic influences on dependence may be mediated by smoking intensity and related behaviors rather than acting as direct determinants^32,33^. Academic performance has also been more commonly conceptualized as an outcome of smoking behavior rather than a primary driver of dependence^34^.

Psychological factors provide additional context for these findings. Conditions such as stress, anxiety, and depression have been consistently associated with higher nicotine dependence^30^. Although these variables were not directly measured in the present study, they may represent underlying pathways linking self-esteem and smoking behavior. Future research incorporating broader mental health measures may help clarify these relationships and improve understanding of the mechanisms underlying nicotine dependence.

### Strengths and limitations

This study has several strengths, including a relatively large sample from multiple institutions and the use of validated instruments (FTND and RSES). The application of multivariable regression allowed adjustment for potential confounders, strengthening the validity of the findings.

However, several limitations should be considered. The cross-sectional design restricts causal inference and precludes establishing temporality between self-esteem and nicotine dependence. Self-reported smoking history and scale-based measures may introduce recall and social desirability bias, as well as potential misclassification of exposure and outcome variables. The university-based sampling frame, characterized by a predominance of males and participants from governmental universities, may limit the generalizability of findings to broader populations. Furthermore, residual confounding may arise from unmeasured psychosocial factors, including stress, anxiety, depression, peer smoking, and other substance use.

Overall, the findings indicate that nicotine dependence is associated with smoking history and self-esteem, highlighting the relevance of psychosocial factors in understanding nicotine dependence in this population.

## CONCLUSIONS

This study demonstrates an association between nicotine dependence and self-esteem among respiratory therapy students in Saudi Arabia. A longer history of smoking was associated with higher dependence, while higher self-esteem was associated with lower dependence. These findings contribute to the understanding of behavioral and psychosocial factors related to nicotine dependence in this population. Further research, particularly longitudinal and interventional studies, is needed to clarify causal pathways and better understand these relationships.

## Data Availability

Individual participant data that underlie the results in the article, after deidentification (text, tables, and appendices), will be made available from the corresponding author upon request. Data will be made immediately available to anyone who wishes to access the data, for any purpose, following publication. Data will be available indefinitely.
